# Image-based explainable artificial intelligence accurately identifies myelodysplastic neoplasms beyond conventional signs of dysplasia

**DOI:** 10.1038/s41698-025-01222-y

**Published:** 2025-12-11

**Authors:** Jan-Niklas Eckardt, Ishan Srivastava, Freya Schulze, Susann Winter, Tim Schmittmann, Sebastian Riechert, Martin M. K. Schneider, Lukas Reichel, Miriam Eva Helena Gediga, Katja Sockel, Anas Shekh Sulaiman, Christoph Röllig, Frank Kroschinsky, Anne-Marie Asemissen, Christian Pohlkamp, Torsten Haferlach, Martin Bornhäuser, Karsten Wendt, Jan Moritz Middeke

**Affiliations:** 1 Department of Internal Medicine I, University Hospital Carl Gustav Carus, TUD Dresden University of Technology, Dresden, Germany; 2 Else Kröner Fresenius Center for Digital Health, TUD Dresden University of Technology, Dresden, Germany; 3 Institute of Software and Multimedia Technology, TUD Dresden University of Technology, Dresden, Germany; 4 Department of Hematology, Oncology and Bone Marrow Transplantation with Section of Pneumology, University Medical Center Hamburg-Eppendorf, Hamburg, Germany; 5 Munich Leukemia Laboratory, Munich, Germany; 6 German Cancer Consortium (DKTK), Partner Site Dresden, and German Cancer Research Center (DKFZ), Heidelberg, Germany; 7 National Center for Tumor Diseases (NCT), NCT/UCC Dresden, a partnership between DKFZ, Faculty of Medicine and University Hospital Carl Gustav Carus, TUD Dresden University of Technology, and Helmholtz-Zentrum Dresden-Rossendorf (HZDR), Dresden, Germany

**Keywords:** Myelodysplastic syndrome, Myelodysplastic syndrome

## Abstract

Cytomorphological assessment of bone marrow smears (BMS) is essential in the diagnosis of myelodysplastic neoplasms (MDS), yet manual evaluation is prone to inter-observer variability. We trained end-to-end deep learning models to distinguish between MDS, acute myeloid leukemia, and bone marrow donor BMS with high accuracy in internal tests and external validation. Occlusion sensitivity mapping revealed the high importance of nuclear structures beyond canonical dysplasia, demonstrating accurate, interpretable MDS detection without labor-intensive cell-level annotation.

Myelodysplastic neoplasms (MDS) encompass clonal myeloid malignancies that are characterized by ineffective hematopoiesis, cytopenia, myelodysplasia, and recurrent genetic events. Accurate cytomorphologic evaluation of the bone marrow remains crucial for the initial diagnosis, response assessment, and detection of disease transformation to acute myeloid leukemia (AML). While counting myeloblasts is rather straightforward, signs of dysplasia are more subtle and their accurate identification requires experienced investigators. Still, detection is often challenging, time- and cost-intensive, prone to inter-observer variability (even for seasoned morphologists)^1,2^, and shows discrepancies between site and central review^3^. Deep learning (DL), especially convolutional neural nets (CNN), excel in image classification tasks, and have recently been used for bone marrow morphology assessment^4–9^. In this study, we used an end-to-end DL system to accurately differentiate between MDS, AML, and bone marrow donor samples based on regions of interest in bone marrow smears, without the need for manually labeling cells or dysplastic morphologies.

We extended our previously described DL pipeline^4,5^ to delineate MDS (*n* = 463), AML (*n* = 1301), and donors (*n* = 236). Based on case-level diagnoses, pre-treatment regions of interest of bone marrow smears from initial diagnosis were labeled with either “MDS”, “AML”, or “donor”. Importantly, no cell-level manual labeling was performed. We trained multiple models for binary classifications, i.e., MDS vs. AML and MDS vs. donors, using six DL architectures with 5-fold cross-validation for every combination. MDS classification performance was externally validated. Baseline characteristics of the MDS patient cohort are shown in Table S1. For the distinction between MDS and donors, we found Densenet-201 to achieve the highest classification performance with an accuracy of 0.98 and a corresponding ROCAUC (area-under-the-curve of the receiver-operating-characteristic) of 0.97 (Table S2; Fig. 1A). Delineating MDS from AML, the best results were obtained using the Squeezenet architecture, resulting in an accuracy of 0.98 and a ROCAUC of 0.99 (Table S2; Fig. 1B). In our AML cohort, 156 patients had AML with myelodysplasia-related changes (AML-MRC). We found our classifier to still be highly accurate even when accounting for MRC status, with an accuracy of 0.95 and 0.98 for AML-MRC and AML without MRC delineated from MDS, respectively (Table S3). The same was true for AML with or without mutated *NPM1*, showing an accuracy of 0.99 and 0.97 delineated from MDS, respectively (Table S4). The training of models (MDS vs. donors and MDS vs. AML) on the high-performance computing (HPC) system required 20 hours to complete each. In external validation, our models achieved an accuracy of 0.99 with a corresponding ROCAUC of 0.98 in distinguishing external MDS from donors (Table S5, Fig. 1C). Delineating external MDS samples from AML, an accuracy of 0.92 was achieved with a ROCAUC of 0.98 (Table S5, Fig. 1D). In subgroup analyses, we trained models to delineate MDS with increased blasts (MDS-IB1/2) from MDS without increased blasts, achieving a ROCAUC of 0.87 (Table S6), and from AML, achieving a ROCAUC of 0.85 (Table S6). Distinguishing MDS-IB1 from MDS-IB2 showed a ROCAUC of 0.80 (Table S6).

**Fig. 1:**
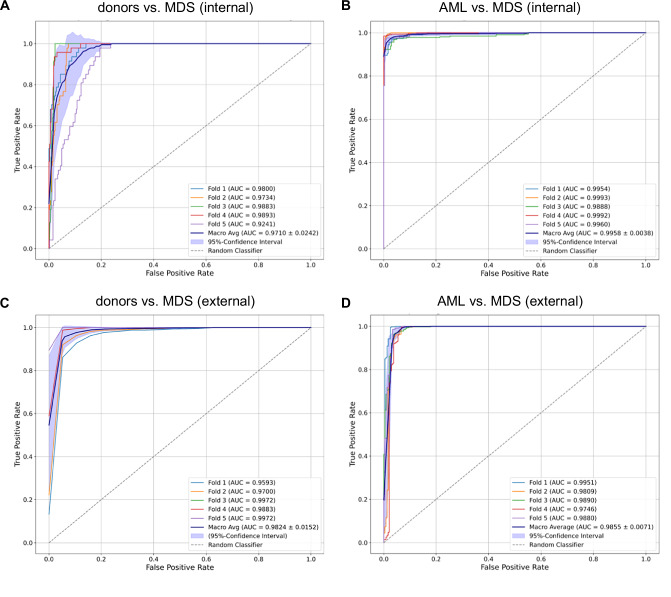
Performance of deep learning models for binary classifications delineating MDS, AML, and donors. The receiver-operating characteristic (ROC) with the corresponding area-under-the-curve (AUC) is shown for the best-performing models for each classification task. Internal validation (**A**, **B**): Only test set results are reported. For MDS vs. donors, the best results were achieved with Densenet-201 (**A**). For MDS vs. AML, the best results were achieved with Squeezenet (**B**). Internal cross-validation was performed with an 80:20 split. Individual run performance (Fold 1-5; graphs in light blue, orange, green, red, and purple) as well as aggregate macro average performance (graph in dark blue) are reported with standard deviation and 95% confidence intervals. External validation (**C**, **D**): Individual run performance regarding MDS vs. donors (**C**) and MDS vs. AML (D) are reported.

To highlight the importance of image areas associated with correct class predictions and thereby identify morphological cues that the network used to delineate MDS, AML, and donors, we used occlusion sensitivity maps (OSM). We found OSM to be cell-specific, indicating that networks focus on cells rather than background or smudge, particularly on granulopoiesis and erythropoiesis as well as megakaryocytes (Fig. 2). High importance was found for defined signs of dysplasia, including altered nuclear morphology such as chromatin clumping, dysfunctional segmentation, or double nuclei. However, at times, high importance in correct class predictions was also found for cells we deemed inconspicuous regarding dysplasia per conventional definition^10^, while neural networks in these cells also mainly focused on the nucleus, sometimes including the perinuclear zone. This indicates more intricate and subtle morphological alterations unquantifiable by human observers. However, other signs of dysplasia, such as hypogranulation, were disregarded by our model. We observed that correct classifications were often given with confidence scores close to 1.0, potentially indicating that the models were confident enough in their decisions to assign a certain class without evaluating all apparent signs of dysplasia (defined or not) or that some signs of dysplasia were simply too rare in the training set to be learned by the models.

**Fig. 2:**
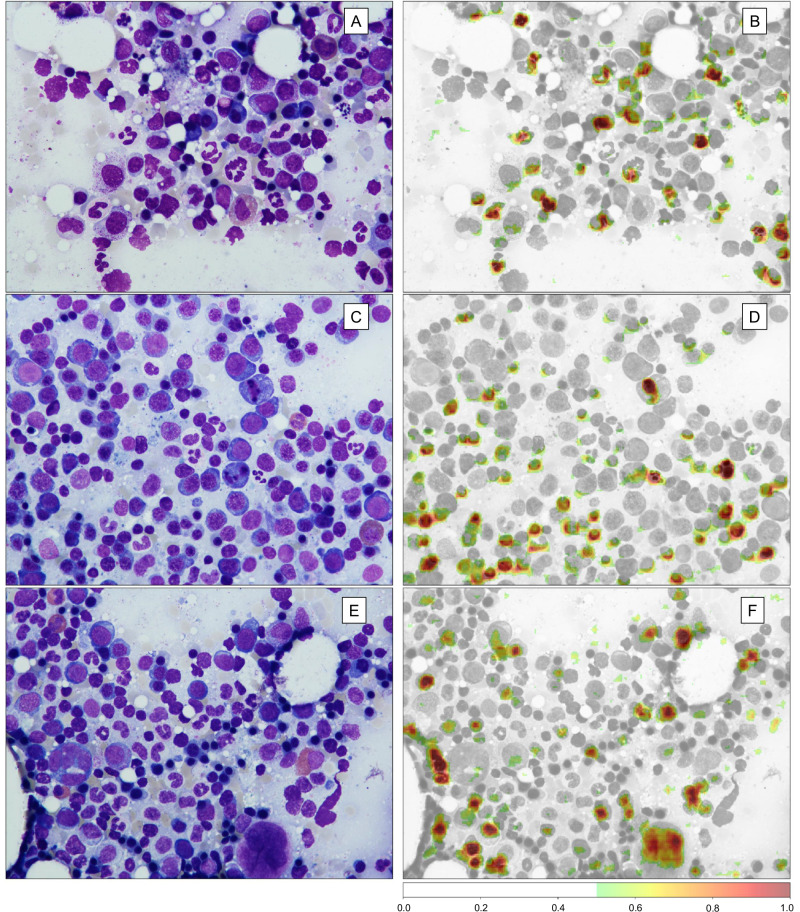
Occlusion Sensitivity Mapping (OSM) highlights image areas with high importance for correct class predictions, enabling output interpretation. OSM iteratively blocks image areas from being evaluated by the deep learning network. If an image area is highly important for classification, the network’s performance will thus drop substantially in the given iteration. Importance is thus standardized between 0 and 1, where values between 0.5 and 1.0 denote high importance, i.e., a sharp drop in classification performance if that image area is blocked (red, see color bar). A standard field of view of bone marrow smears from MDS patients is shown in (**A**, **C**, and **E**). The corresponding OSM is displayed in (**B**, **D**, and **F**), respectively. First, in a proof-of-concept fashion, the networks focus on cells and specifically on nuclei. Background, noise, or smudge are not considered important for classification. Second, high importance is given to erythropoietic and granulopoietic cells as well as megakaryocytes.

Using end-to-end DL, we developed a software framework to distinguish between MDS, AML, and donors with very high accuracy based on bone marrow smears from 2000 individual patients and bone marrow donors. Importantly, we have demonstrated that information abstraction, even in MDS with often subtle morphologies, is feasible using end-to-end learning, in contrast to recent studies in hematology that primarily rely on the generation of cell-level labels^6–9^. Using the latter approach, a bottom-up system has to be devised, where first thousands of labels are required to build a robust classifier, and second, individual cell-level predictions have to be aggregated to generate a diagnosis-level prediction. Apart from being time-consuming and cost-ineffective, the generation of cell-level labels, i.e., the ground truth that many classifiers in hematology currently are based on, is flawed due to substantial classification biases^2^. This bottleneck and pitfall of cell-level labeling can essentially be bypassed by an end-to-end approach using robust region-of-interest-level labels. Similar approaches have yielded favorable results in generating differential counts^11^ as well as *NPM1* and *FLT3-ITD* mutation status prediction^12^.

With respect to explainability, DL is often referred to as a ‘black box’. Using OSM not only enables an internal proof-of-concept, but also provides additional information to the human observer, as novel features that are important for prediction can be investigated that otherwise would elude the human eye. Interestingly, our classifiers focused on nuclei not only in dysplastic cells, but also in cells that we did not deem to be morphologically suspicious of dysplasia. Potentially, this alludes to digital biomarkers in MDS distinct from classical signs of dysplasia. Future work will focus on correlating saliency maps with genetic alterations and/or gene expression in MDS. While certain molecular alterations have already been linked to certain morphologies, such as mutated *SF3B1* in MDS with ring sideroblasts, CNNs can potentially be used to identify novel gene-morphology links^13^.

Our study is limited by several factors. As is the case for most recent studies of computer vision in (hemato-)pathology, our analysis is based on retrospective data. While external validation confirmed high classification accuracy, prospective validation is still warranted. While manual selection of regions of interest mirrors workflows in clinical routine, it introduces the necessity of expert input, and thus manual labor, into the workflow, and potentially may introduce bias, as classification performance may drop if suboptimal image areas are provided. In our study, we differentiated only between AML, MDS, and donors in a binary way. Still, some dysplastic morphologies can also be present to a certain degree in non-malignant disorders such as congenital syndromes, nutritional deficiencies, infectious disease, and drug- or toxin-mediated bone marrow damage. To increase routine applicability, future work will also focus on acquiring image data from reactive and non-neoplastic specimens exhibiting signs of dysplasia in order to make our classifier more versatile and applicable in clinical routine. To this end, multi-class predictions in a single classifier are a viable alternative to binary classifications as more diagnostic classes are added, ensuring that one single model may practically inform diagnostic decision-making rather than having to apply multiple models on the same slide. Further, the updated WHO^14^ and ICC classifications^15^ confirm or introduce several genetically defined subtypes of AML and MDS, MDS with increased blasts, as well as (in the case of the ICC) AML/MDS overlap syndrome. Given the rarity of some of these subtypes on one side and, in computer vision terms, relative scarcity of training data in our sample on the other, training computer vision models to delineate these subtypes was currently not feasible, but is planned for future multicenter studies. While several pathology foundation models have been introduced recently^16^, neither have they been trained on large corpora of bone marrow smear images nor have they been systematically evaluated for bone marrow morphology or MDS classification tasks, providing another avenue for further studies.

In summary, we developed a DL framework trained on patient and donor samples, achieving high accuracies in our internal test set and external validation in distinguishing between MDS, AML, and donors.

## Methods

### Data sets

We identified 463 MDS patients who had been previously diagnosed and treated at the University Hospital Dresden, Germany. The first control group was comprised of 1301 AML patients that had been diagnosed and treated under the auspices of the multicenter German Study Alliance Leukemia (SAL) within the following previously reported multicenter trials: AML96 [NCT00180115], AML2003 [NCT00180102], AML60+ [NCT00180167], and SORAML [NCT00893373]. Patients were eligible upon diagnosis of MDS or AML, age ≥18 years, and available biomaterial at initial diagnosis, including bone marrow smears. The second control group consisted of 236 bone marrow samples from healthy bone marrow donors who underwent allogeneic bone marrow donation at our center. An additional external validation cohort was obtained from the Munich Leukemia Laboratory (MLL), Munich, Germany, consisting of 50 MDS patients evaluated in conjunction with held-out test sets of AML and donor samples from our internal cohort. Prior to analysis, written informed consent was obtained from all patients and donors according to the revised Declaration of Helsinki^17^. All studies were approved by the Institutional Review Board of the TUD Dresden University of Technology (EK 98032010 and EK 289112008).

### Image digitization

Pre-treatment bone marrow smears from the initial diagnosis were used within this study. Staining of MDS, AML, and donor bone marrow smears was performed from anticoagulated bone marrow with the May-Gruenwald-Giemsa method^10^. Disease class labels were derived from case-level diagnostics, including cytomorphology, histology, cytogenetics, and molecular genetics, previously documented for each case during routine diagnostics or as part of the respective clinical trial. Using a Pannoramic SCAN II (3DHISTECH), we obtained high-resolution whole slide images with a 20x objective and a 1.6x C-mount adapter, yielding a resolution of 0.20353 µm/pixel. For every AML patient and bone marrow donor, we selected a single region viewed at 50x digital magnification in SlideViewer (3DHISTECH) and exported the image. We assumed that subtle signs of dysplasia would not be fully captured in one field of view alone. Therefore, for each MDS patient, we selected four regions of interest from the whole-slide image during training and testing on our internal data, viewed each at 50× digital magnification, and exported them as images for analysis. For external validation, 10 regions of interest were selected for MDS slides to accommodate the relatively smaller sample size of the validation cohort. Evaluation of bone marrow smears and potential regions of interest was performed by board-certified hematologists, mirroring clinical routine in selecting image areas according to stain quality, cellularity, even cell distribution, and avoidance of overlapping or clumping cells, presence of bone marrow spicules (rather than peripheral blood), absence of artifacts, and digital image quality.

### Deep learning

#### End-to-end prediction on regions of interest from bone marrow slides

We extended our previously described DL pipeline^4,5^ for binary predictions on regions of interest on bone marrow smears for the delineation of MDS, AML, and donors. Based on case-level diagnoses, images were labeled with either “MDS”, “AML”, or “donor”. Importantly, no cell-level manual labeling was performed. The pipeline was adapted to evaluate cases in a binary fashion, i.e., MDS vs. AML and MDS vs. donors. Potentially, imbalanced training data can bias a classifier towards the predominant class. Considering the imbalances between the data sets (n = 463 samples for MDS with 4 images per patient, resulting in 1852 MDS images in total; n = 1301 samples for AML with 1 image per patient; *n* = 236 samples per donor with 1 image per donor), we used image augmentation techniques, such as random sized cropping, color shifting and linear transformations, to balance the data sets for each binary classification task. For all binary classifications, a 5-fold internal cross-validation was used, i.e., a train-test split of 80:20. Cases that were used for model training were strictly separated from cases that were used for testing. In DL, determination of an optimal model cannot be done a priori, but rather has to be evaluated given the specific use case, data set, and model architecture. Hence, we evaluated six recently introduced DL architectures for computer vision, including ResNet-18/34/50/101/152^18^, ResNeXt-50_32x4d/101_32x8d^19^, Wide-ResNet-50/101^20^, DenseNet-121/161/169/201^21^, ShuffleNet v2_x0_5/v2_x1_02^22^, and SqueezeNet v1.1^23^. All DL models were pre-trained on ImageNet data^24^. The final architecture for each model was determined using automated hyperparameter optimization with the Optuna framework^25^. DL models were implemented in Python using the PyTorch framework. Computations were performed using the high-performance computing (HPC) cluster of the TUD Dresden University of Technology.

### Performance evaluation

Recall (*syn*.: sensitivity), precision (*syn*.: positive predictive value), and accuracy were used to evaluate classification performances. Recall is defined as the fraction of all positive predictions among all relevant events, and precision is defined as the fraction of true positives among all positive predictions. Further, the area-under-the-curve (AUC) was determined for the receiver-operating-characteristic (ROC). All metrics are reported for each binary classification for the internal test sets as well as for the external validation cohort with 95% confidence intervals.

### Explainability of classifications via occlusion sensitivity maps

In order to derive morphologies that were identified by the neural networks for class predictions, methods of model explainability can be employed. Generally, explainability of computer vision models refers to techniques that visualize image areas of high importance for correct classifications, thereby allowing the identification of image areas or objects that confirm that the model learnt relevant features or enable identification of digital biomarkers by domain experts. In our study, to highlight image areas the network assigned high importance to and thereby identify morphological cues the network used to delineate MDS, AML, and donors, we used occlusion sensitivity maps (OSM)^26^. In OSM, random image areas are iteratively blocked from the view of the CNN, and classification performance is measured. If the blocked image area is highly relevant for accurate classification, model performance will drop accordingly. This process is repeated for the entire image. Thus, image areas that are crucial for accurate predictions are highlighted so that morphologies that prompt the CNN classifier to predict a label can be evaluated and interpreted. The importance of image areas was scaled between 0 and 1. For visualizations, a threshold of 0.5 was used, and only image areas between confidence scores of 0.5 to 1.0 are highlighted and referred to as areas of high importance. Areas of high or low importance for class predictions on region of interest images were subsequently assessed by at least three independent board-certified hematologists per image to identify relevant morphologies highlighted or missed by OSM.

## Supplementary information

Supplementary information

## Data Availability

Code is available under [https://github.com/Ai-in-Cancer-org/MDS_pipeline/] Example ROIs of MDS and AML patients are available under 10.6084/m9.figshare.30196588. Additional data that support the findings of this study are available from the corresponding author upon reasonable request.
