# Supplementary material for: Image-based explainable artificial intelligence accurately identifies myelodysplastic neoplasms beyond conventional signs of dysplasia
Source: NPJ Precis Oncol. 2025 Dec 11;10:26. doi: 10.1038/s41698-025-01222-y (PMC12808678; doi:10.1038/s41698-025-01222-y)
Supplement: Supplementary file 1 — Supplementary information [file 41698_2025_1222_MOESM1_ESM.docx]

**Table S1 MDS patient characteristics**

| **Parameter** |  |
| --- | --- |
| **N** | 463 |
| **Age in years, median (IQR)** | 66 (18-89) |
| **Sex, %** |  |
| Male | 59 |
| Female | 41 |
| **MDS type (WHO 2022), %** |  |
| MDS-5q | 10 |
| with *SF3B1* mutation | 0.1 |
| with *TP53* mutation | 0.1 |
| MDS bi*TP53* | 0.2 |
| MDS *SF3B1* | 8.8 |
| MDS-LB | 29.3 |
| MDS, hypoplastic | 2.7 |
| MDS-IB1 | 15.6 |
| MDS-IB2 | 20 |
| MDS with fibrosis | 1.3 |
| MDS/MPN-RS-T | 0.5 |
| CMML-1 | 1.2 |
| CMML-2 | 9.8 |
| **IPSS-R, %** |  |
| Very low risk | 6.4 |
| Low risk | 21.6 |
| Intermediate risk | 38.7 |
| High risk | 22.3 |
| Very high risk | 10.9 |
| **Blood count** |  |
| WBC in GPt/l, median (IQR) | 3.58 (0.57-91.1) |
| Hb in g/dl, median (IQR) | 9.9 (4.4-15.6) |
| Plt in GPt/l, median (IQR) | 96 (3-1531) |
| PB blasts in %, median (IQR) | 0 (0-15) |
| BM blasts in %, median (IQR) | 5.5 (0-26.0) |

*BM* bone marrow, *CMML-1/2* chronic myelomonocytic leukemia subgroup 1/2, *Hb* hemoglobin, *MDS* myelodysplastic neoplasm, *MDS biTP53* MDS with biallelic *TP53* inactivation, *MDS-5q* MDS with low blasts and isolated 5q deletion, *MDS-IB1/2* MDS with increased blasts 1/2, *MDS-LB* MDS with low blasts, *MDS/MPN-RS-T* myelodysplastic/myeloproliferative neoplasm with ring sideroblasts and thrombocytosis, *MDS-SF3B1* MDS with low blasts and *SF3B1* mutation, *N* number, *PB* peripheral blood, *Plt* platelet count, *WBC* white blood cell count.

**Table S2 Test set performance for binary ROI-level classifications**

|  | **MDS vs. donors** | | **MDS vs. AML** | |
| --- | --- | --- | --- | --- |
| DL architecture | Densenet-201 | | Squeezenet v1.1 | |
| Accuracy  [95%-CI] | 0.97791  [0.9597 - 0.9961] | | 0.98072  [0.9710 - 0.9904] | |
|  | **MDS** | **donors** | **MDS** | **AML** |
| Precision  [95%-CI] | 0.9973  [0.9951 - 0.9995] | 0.8547  [0.737 - 0.973] | 0.97065  [0.9526 - 0.9888] | 0.98118  **[0.9639 - 0.9985]** |
|  | **MDS** | **donors** | **MDS** | **AML** |
| Recall  [95%-CI] | 0.9775  [0.9566 - 0.9984] | 0.9787  [0.9599 - 0.9975] | 0.98180  [0.9642 - 0.9994] | 0.98030  [0.9648 - 0.9958] |
| ROCAUC  [Fold-wise range] | 0.9708  [0.9241 - 0.9893] | | 0.9958  [0.9888 - 0.9993] | |

*CI* confidence interval, *AML* acute myeloid leukemia, *DL* deep learning, *MDS* myelodysplastic neoplasm, *ROCAUC* area-under-the-curve of the receiver-operating-characteristic, *ROI* region of interest.

**Table S3 Test set ROI-based performance of the MDS vs. AML classifier regarding AML with or without myelodysplasia-related changes (MRC)**

| **Group** | **Total test set ROIs** | **TP** | **FN** | **Accuracy** |
| --- | --- | --- | --- | --- |
| AML-MRC | 20 | 19 | 1 | 0.950 |
| Non-MRC AML | 226 | 221 | 5 | 0.978 |
| MDS | 392 | 375 | 17 | 0.957 |

True positives (TP) and false negatives (FN) are calculated assuming positive labels (1) as AML and negative labels (0) as MDS.

**Table S4 Test set ROI-based performance of the MDS vs. AML classifier regarding AML with or without *NPM1* mutations**

| **Group** | **Total test set ROIs** | **TP** | **FN** | **Accuracy** |
| --- | --- | --- | --- | --- |
| NPM1+ AML | 70 | 69 | 1 | 0.986 |
| NPM1- AML | 176 | 171 | 5 | 0.972 |
| MDS | 392 | 375 | 17 | 0.957 |

True positives (TP) and false negatives (FN) are calculated assuming positive labels (1) as AML and negative labels (0) as MDS.

**Table S5 Model performance on external validation set**

|  | **MDS (MLL cohort) vs. donors** | | **MDS (MLL cohort) vs. AML** | |
| --- | --- | --- | --- | --- |
| DL architecture | Densenet-201 | | Squeezenet v1.1 | |
| Accuracy  [95%-CI] | 0.9902  [0.9830 - 0.9974] | | 0.92104  [0.8919 - 0.9501] | |
|  | **MDS** | **donors** | **MDS** | **AML** |
| Precision  [95%-CI] | 0.9925  [0.9873 - 0.9977] | 0.9852  [0.9774 - 0.9930] | 0.91418  [0.891 - 0.937] | 0.94668  [0.860 - 1.0] |
|  | **MDS** | **donors** | **MDS** | **AML** |
| Recall  [95%-CI] | 0.9970  [0.9949 - 0.9991] | 0.9938  [0.9810 - 1.0] | 0.97516  [0.932 - 1.0] | 0.80834  [0.751 - 0.866] |
| ROCAUC  [Fold-wise range] | 0.9823  [0.9593 - 0.9972] | | 0.9847  [0.9746 - 0.9951] | |

*CI* confidence interval, *AML* acute myeloid leukemia, *MDS* myelodysplastic neoplasm, *MLL* Munich Leukemia Laboratory, *ROCAUC* area-under-the-curve of the receiver-operating-characteristic.

**Table S6 Classification performance for subgroup analyses regarding MDS with or without increased blasts (IB) and vs. AML with the best performing model for each classification**

| **Subgroup analyses** | **Number of patients and ROIs** | **Accuracy** | **ROCAUC  [Fold-wise range]** | **Model** |
| --- | --- | --- | --- | --- |
| MDS-non-IB1/2 vs.  MDS-IB1/2 | Patients: 274 vs. 207  ROIs: 1194 vs. 860 | 78.13% | 0.871 [0.860 - 0.875] | ResNet 34 |
| MDS-IB 1/2 vs. AML | Patients: 207 vs. 1301  ROIs: 860 vs. 1301 | 77.38% | 0.849 [0.840 - 0.856] | ResNet 34 |
| MDS-IB1 vs. MDS-IB2 | Patients: 116 vs. 91  ROIs: 490 vs. 370 | 71.44% | 0.802 [0.795 - 0.810] | Squeezenet v1.1 |

*ROCAUC* area-under-the-curve of the receiver-operating-characteristic, *ROI* region of interest.
